# Supplementary figures and images for: Evolutionary history of calcium-sensing receptors unveils hyper/hypocalcemia-causing mutations
Source: PLoS Comput Biol. 2024 Nov 12;20(11):e1012591. doi: 10.1371/journal.pcbi.1012591 (PMC11584096; doi:10.1371/journal.pcbi.1012591)

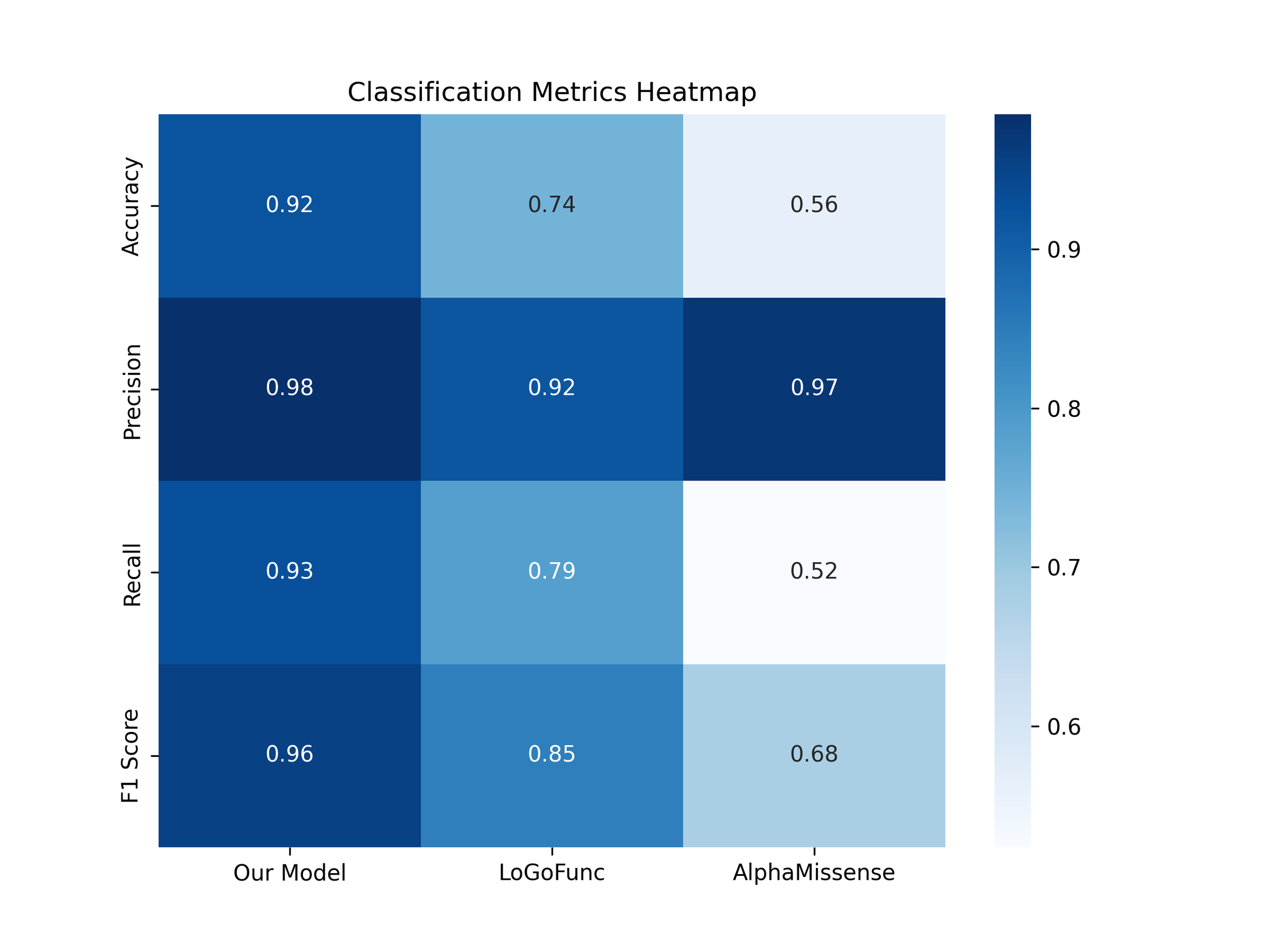

Supplement: S1 Fig — The performance of three prediction models—Our Model, LoGoFunc, and AlphaMissense—across four key metrics: Accuracy, Precision, Recall, and F1 Score are shown. The values are color-coded, with darker shades indicating higher performance. Our Model demonstrates superior performance in all metrics compared to the other tools, highlighting its enhanced predictive capability for classifying CASR gene mutations. (TIF) [file pcbi.1012591.s001.tif]

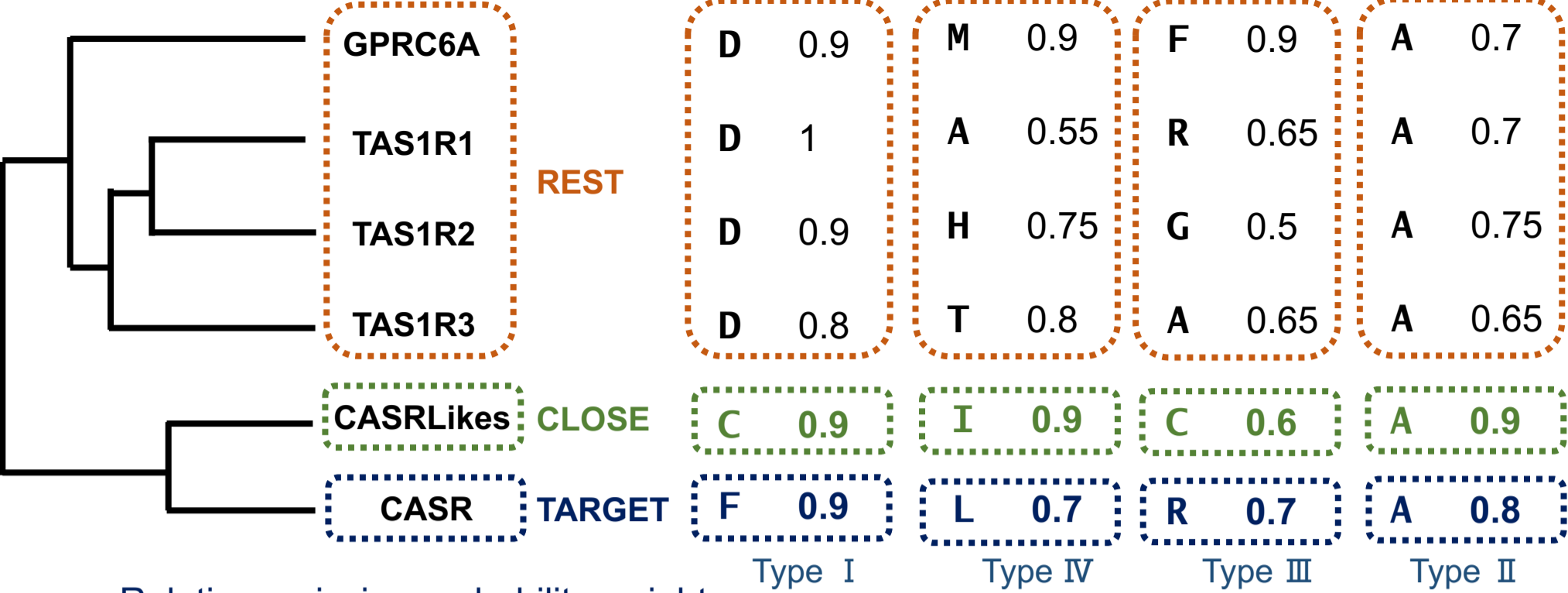

Relative emission probability weight for CASR ssHMM:

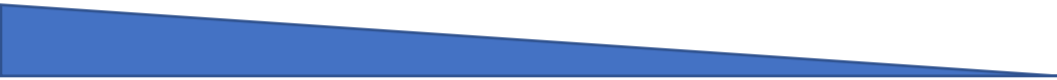

Supplement: S2 Fig — We considered different types to weight emission probabilities of profile HMMs. (PDF) [file pcbi.1012591.s002.pdf]
