# Supplementary material for: Evolutionary history of calcium-sensing receptors unveils hyper/hypocalcemia-causing mutations
Source: PLoS Comput Biol. 2024 Nov 12;20(11):e1012591. doi: 10.1371/journal.pcbi.1012591 (PMC11584096; doi:10.1371/journal.pcbi.1012591)
Supplement: S1 Table — (PDF) [file pcbi.1012591.s003.pdf]

S1 Table: **Comparison of accuracy and F1 score between tools**

Tool	Accuracy	F1 Score
Our Approach	0.77	0.83
GOF/LOF	0.77	0.85
